# Study on the Simulation of Biosensors Based on Stacked Source Trench Gate TFET

**DOI:** 10.3390/nano13030531

**Published:** 2023-01-28

**Authors:** Chen Chong, Hongxia Liu, Shougang Du, Shulong Wang, Hao Zhang

**Affiliations:** Key Laboratory for Wide Band Gap Semiconductor Materials and Devices of Education Ministry, School of Microelectronics, Xidian University, Xi’an 710071, China

**Keywords:** dielectric modulated stacked source trench gate tunnel FET (DM-SSTGTFET), biosensor, sensitivity

## Abstract

In order to detect biomolecules, a biosensor based on a dielectric-modulated stacked source trench gate tunnel field effect transistor (DM-SSTGTFET) is proposed. The stacked source structure can simultaneously make the on-state current higher and the off-state current lower. The trench gate structure will increase the tunneling area and tunneling probability. Technology computer-aided design (TCAD) is used for the sensitivity study of the proposed structured biosensor. The results show that the current sensitivity of the DM-SSTGTFET biosensor can be as high as 10^8^, the threshold voltage sensitivity can reach 0.46 V and the subthreshold swing sensitivity can reach 0.8. As a result of its high sensitivity and low power consumption, the proposed biosensor has highly promising prospects.

## 1. Introduction

In recent years, various sensors have been extensively studied, such as piezoelectric nanosensors [1,2], magnetic sensors [3,4], PH sensors [5], gas sensors [6], CNT nano-resonators [7], nanowire sensors [8] and biosensors [9,10]. Among them, biosensors have broad application prospects in clinical diagnosis, industrial control, drug analysis, biotechnology, biochips and other research in the national economy.

Dielectric modulation is to etch the nanogap in the gate oxide layer of the device and then fill it with biomolecules, thereby realizing the role of a biosensor. This is the simplest and most convenient way to realize a biosensor. Therefore, dielectric modulation biosensors have been extensively studied by many researchers [11,12,13]. The band–band tunneling conduction mechanism of TFET can break through the limitation of thermionic emission; therefore, it can reach a sub-threshold swing below 60 mV/dec [14,15,16,17]. Hence, the study of dielectrically modulated TFET biosensors has aroused the interest of many researchers [18,19,20,21]. Rakhi studied the effect of partial hybridization in a dual-gate TFET biosensor on the static electricity and current of the device. As a result, unlike nanogap embedded DM-FETs, TFET-based sensors have no scaling issues [22]. However, the proposed double-gate structure is simple and inconvenient for the filling of biomolecules. In 2019, Rupam compared the sensitivity of a circular gate TFET and a uniform gate heterojunction TFET and found that the sensitivity of the circular gate TFET is higher and the sensitivity of the TFET depends on the position of the biomolecule relative to the tunnel junction [23]. However, the circular gate is difficult to achieve in process realization. In the same year, Sunny studied the junctionless double-gate TFET biosensor and found that its current sensitivity was very high [24]. However, since the size of biomolecules was not as small as 1 nm, the smallest size of nanometer thickness used in this article is 5 nm. In 2021, Gagritee compared single-gate TFET and dual-gate TFET biosensors and found that the dual-gate TFET biosensor had higher sensitivity and development potential [25]. However, the proposed structure is not as convenient as the trench gate structure for the filling of biomolecules.

In this paper, a dual-stack, heterogeneous source, trench gate structure TFET is adopted. The dual-source structure can make the on-state current of the device larger, the stacked heterogeneous source structure allows the device to obtain both high on-state drain current and low off-state drain current at the same time and the trench gate structure makes the filling of biomolecules more convenient. Section 2 describes the basic structure, working principle and simulation method of the proposed biosensor. Section 3 shows the changes in the sensitivity of the biosensor under different biomolecules, different nanogap thicknesses, different charges and different filling profiles. Section 4 concludes the research findings from the performed investigations.

## 2. Device Structure and Simulation Method

### 2.1. Device Structure

**Figure 1:**
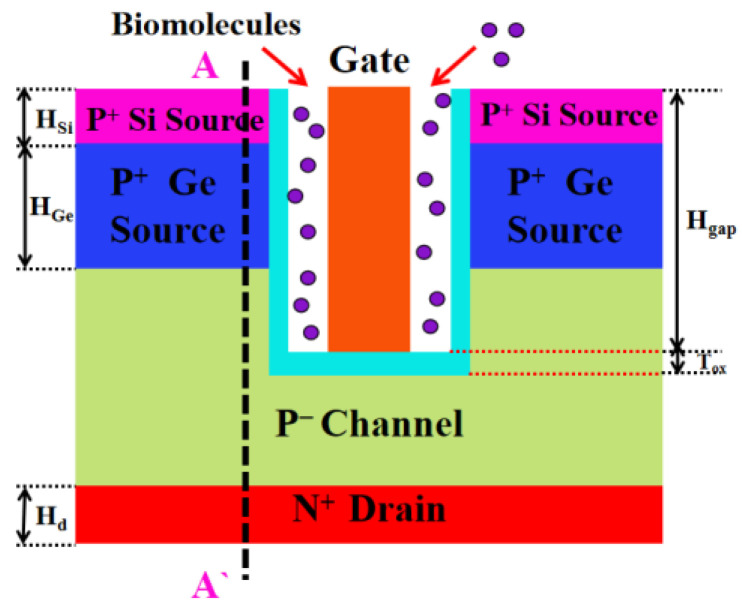
Schematic cross-sectional view of DM-SSTGTFET biosensor.

A cross-sectional view of a DM-SSTGTFET biosensor is shown in Figure 1. The channel and drain region are made of silicon. The source region is formed by stacking two layers of material. The upper layer material is silicon and the lower layer material is germanium. The gate oxide layer adopts HfO_2_ and the gate metal work function is 4.4 eV. Nanogaps were etched on both sides of the gate electrode to fill the biomolecules, so as to realize the function of the sensor. The detailed parameters are shown in Table 1.

**Figure 2:**
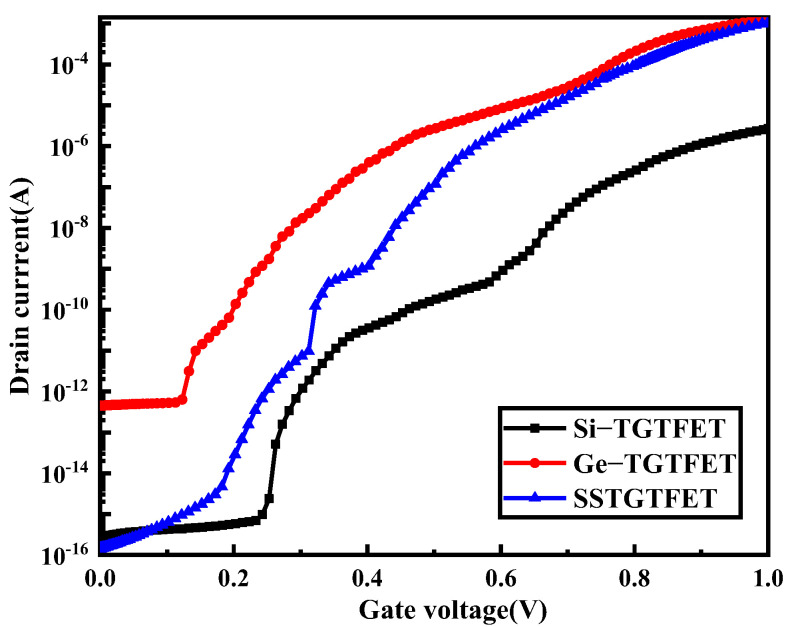
Transfer characteristics for Si−TFET, Ge−TFET and SSTGTFET at V_d_ = 1 V.

In this paper, the transistor with a silicon source material is called Si-TGTFET, and the transistor with a germanium source material is called Ge-TGTFET. Figure 2 shows the transfer curves of three transistors with different source materials and the same other structural parameters. It can clearly be seen that the off-state drain current of the silicon-TGTFET is very small, with a value of about 10^−16^ A. At the same time, the on-state current is also very small, about 10^−6^ A. The off-state drain current of Ge-TGTFET is about 10^−12^ A, and the on-state current is also about 5 × 10^−4^ A. Generally, the smaller the off-state drain current, the smaller the static power consumption of the transistor. The greater the on-state current, the stronger the driving capability of the transistor. Therefore, the lower the off-state drain current and the larger the on-state current, the better the performance of the transistor. In this paper, SSTGTFET combines the advantages of Si-TGTFET and Ge-TGTFET, which have both a small off-state drain current and a large on-state current.

**Figure 3:**
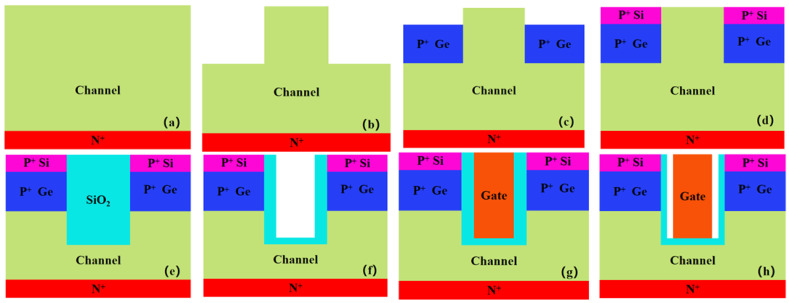

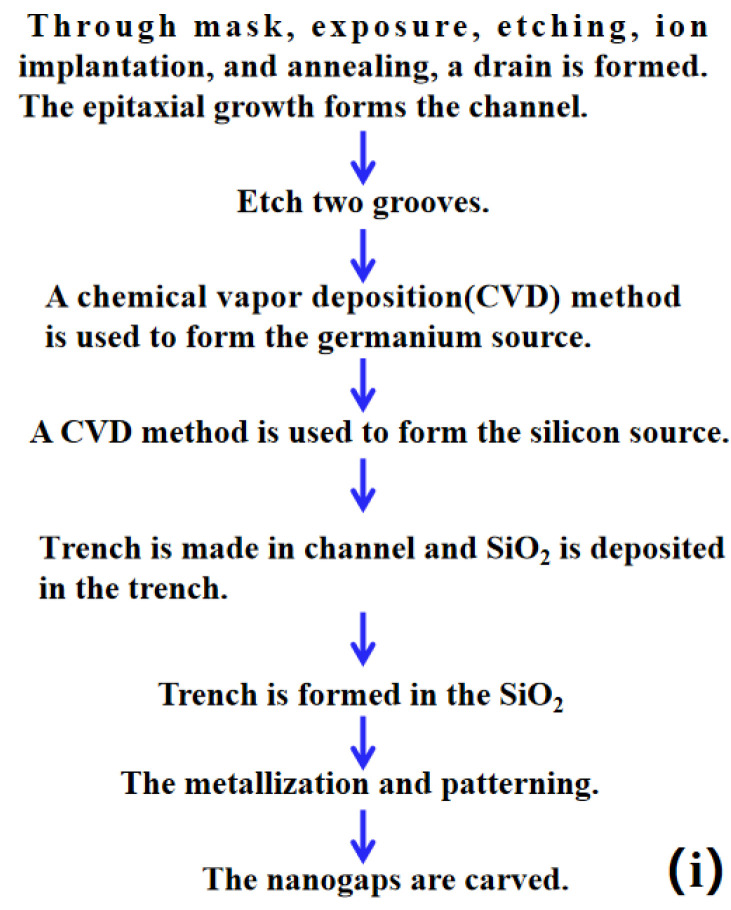
(**a**–**i**) Tentative fabrication flow for a DM-SSTGTFET biosensor.

The preparation process of EETGTFET is similar to the structure in Ref. [26]. Figure 3 shows the process flow of DM-SSTGTFET. Figure 3i is a brief description of Figure 3a–h.

### 2.2. Simulation Method

In this paper, computer-aided simulation is used to simulate DM-SSTGTFET. Sentaurus TCAD software was used to conduct simulations in this paper, which is often used in semiconductor device simulations. The software contains many physical models of semiconductor process; through these models one can simulate the semiconductor electrical characteristics, so as to achieve the function of computer-aided design. In order to obtain more realistic results, suitable physical models were used and the rest of the general models used the default model.

The carrier statistical model adopts the Fermi statistical distribution because of the heavy doping in the proposed structure. At the same time, since heavy doping will make the band gap of the semiconductor smaller, the band gap narrowing model was also used. The recombination model adopts SRH recombination in indirect recombination. The tunneling model uses a non-local tunneling model. In this model, the electric field in the tunneling path is variable, which is in line with the actual situation. This tunneling model adopts the Kane model, in which the band-to-band tunneling rate is [27]:

(1) $$G_{BTBT}=A{\left(\frac{E}{E_{0}}\right)}^{P}\exp\left(-\frac{B}{E_{0}}\right)\;$$

where *E*_0_ = 1 V/cm and *P* = 2.5. In silicon, *A* = 1.63 × 10^14^/cm^3^ s and *B* = 1.47 × 10^7^ V/cm. In germanium, *A* = 1.46 × 10^14^/cm^3^ s and *B* = 3.59 × 10^6^ V/cm. (The values of *A* and *B* are obtained through parameter calibration [28]).

Since the nanogap is filled with organic biomolecules, the biomolecule recombination model is added to this area. The bimolecular recombination rate is given by:

(2) $$R_{bimolec}=\gamma \cdot \frac{q}{\varepsilon_{0}\varepsilon_{r}}\left(\mu_{n}+\mu_{p}\right)\left(np-n_{i,eff}^{2}\frac{n_{se}}{n_{se}^{eq}}\right)\;$$

where *γ* is a prefactor for the singlet exciton. *q* is the elementary charge. *ε*_0_ and *ε_r_* denote the free space and relative permittivities, respectively. *n_se_* is the singlet exciton density. $n_{se}^{eq}$ denotes the singlet–exciton equilibrium density. Electron and hole mobilities are given by *μ_n_* and *μ_p_*, accordingly. *n*, *p* and *n_i,eff_* describe the electron, hole and effective intrinsic density, respectively.

In this paper, seven kinds of small biomolecules with different dielectric (1.6, 2.6, 5, 8, 10, 14 and 23) were filled in nanogap cavities of different thicknesses (5 nm, 7 nm, 9 nm, 11 nm and 13 nm) and were given different amounts of charge to be studied and analyzed when DM-SSTGTFET is in the on-state (*V_d_* = 1 V and *V_g_* = 1 V). Taking the filling of air in the nanogap as a reference, the threshold voltage sensitivity (*S_Vth_*), drain current sensitivity (*S_d_*) and subthreshold swing sensitivity (*S_SS_*) of different biomolecules filling the nanogap are studied. *S_Vth_* is defined as [29]:

(3) $$S_{Vth}=V_{th\left(air\right)}-V_{th\left(bio\right)}\;\;$$

where *V_th_*_(*air*)_ is the threshold voltage of the sensor when the nanogap is filled with air and *V_th_*_(*bio*)_ is the threshold voltage of the sensor when the nanogap is filled with biomolecules.

*S_d_* is expressed as [24]:

(4) $$S_{d}=\frac{I_{on\left(bio\right)}-I_{on\left(air\right)}}{I_{on\left(air\right)}}$$

where *I_on_*_(*air*)_ is the on-state drain current of the sensor when the nanogap is filled with air and *I_on_*_(*bio*)_ is the on-state drain current of the sensor when the nanogap is filled with biomolecules.

The formula for the *S_SS_* is [30]:

(5) $$S_{SS}=\frac{SS_{air}-SS_{bio}}{SS_{air}}$$

where *SS_air_* is the average subthreshold swing of the sensor when the nanogap is filled with air and *SS_bio_* is the average subthreshold swing of the sensor when the nanogap is filled with biomolecules.

## 3. Results and Discussion

### 3.1. Different Biomolecules

Different biomolecules have different dielectric constants. In this paper, the biomolecules with dielectric constants of 1.6, 2.6, 5, 8, 10, 14 and 23 are uriease, biotin, bacteriophage T7, keratin, staphylococcal nuclease, gelatin and egg protein, respectively.

**Figure 4:**
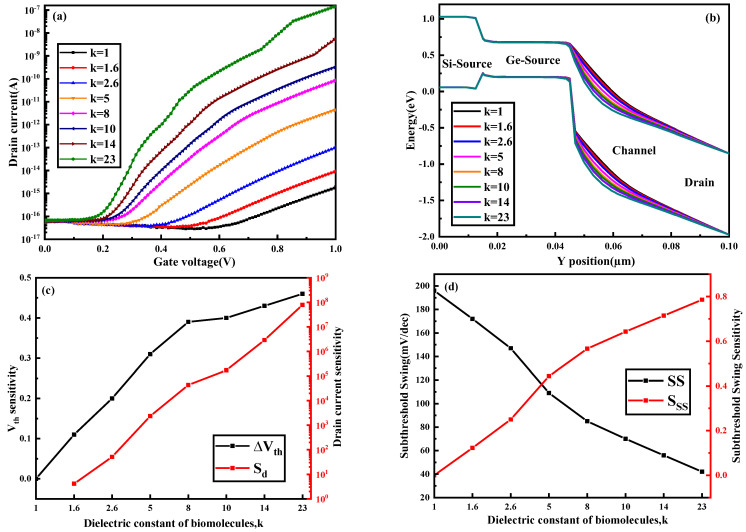
(**a**) Transfer characteristics, (**b**) energy band variation with respect to the *y*−axis, (**c**) *S_Vth_* and *S_d_* and (**d**) *SS* and *S_SS_* of the DM−SSTGTFET biosensor for different values of k at *V_d_* = 0.5 V, *V_g_* = 1 V and *t_c_* = 5 nm.

It can be seen from Figure 2 that as the value of k increases, the transfer curve of the biosensor shifts to the left. At the same time, the threshold voltage of the biosensor decreases and the on-state drain current increases. The energy band diagram in Figure 4b is cut along the AA’ dividing line in Figure 1. It can be seen from Figure 4b that as the value of k increases, the band at the channel bends more severely. This is because the greater the dielectric constant of the biomolecules, the stronger the coupling with the source and channel. The more the band is bent, the easier it is for the biosensor to turn on, and therefore the lower the threshold voltage. At the same time, the more the band is bent, the more electrons and holes tunnel through the band-to-band, so the on-state drain current will be greater. This is consistent with the conclusion shown in Figure 4a. Figure 4c shows that as the value of k increases, the threshold voltage sensitivity and current sensitivity are higher. It can be seen from Figure 4d that as the value of k increases, the subthreshold swing of the biosensor decreases, and at the same time the subthreshold swing sensitivity increases. The subthreshold swing is defined as the amount of change in the gate voltage required to change the drain current ten times. Therefore, the smaller the subthreshold swing, the smaller the power consumption. Only when the k values are 14 and 23, is the subthreshold swing lower than 60 mV/dec, reaching a value that the thermionic emission device cannot reach.

### 3.2. The Influence of Nanogap Thickness on Biosensor

**Figure 5:**
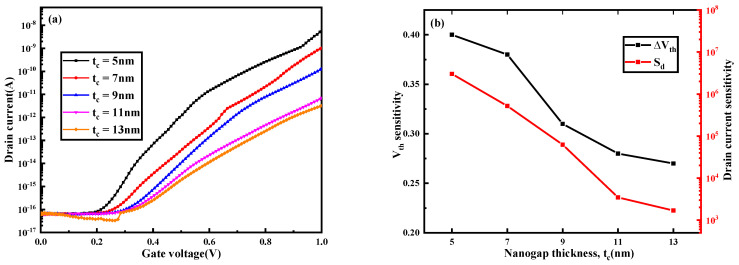

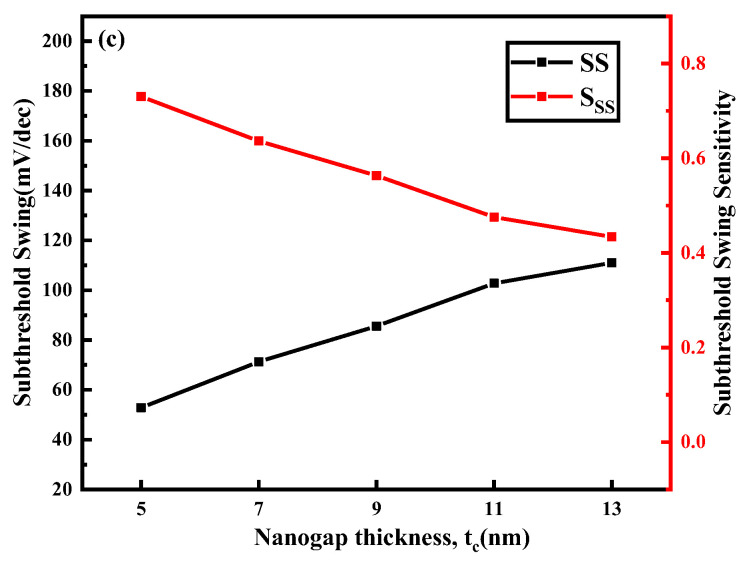
(**a**) Transfer characteristics and (**b**) *S_Vth_* and *S_d_* and (**c**) *SS* and *S_SS_* of the DM−SSTGTFET biosensor for different thicknesses of nanogap at *V_d_* = 0.5 V, *V_g_* = 1 V and k = 14.

The thickness of the nanogap has different effects on the sensitivity of the biosensor. According to the size of the biomolecules, the thicknesses of the nanogaps used in this paper are 5 nm, 7 nm, 9 nm, 11 nm and 13 nm. As the thickness of the nanogap increases, the transfer curve of the device shifts to the left, the threshold voltage sensitivity decreases, the drain current sensitivity decreases, the subthreshold swing increases and the subthreshold swing sensitivity decreases. These can be seen in Figure 5.

**Figure 6:**
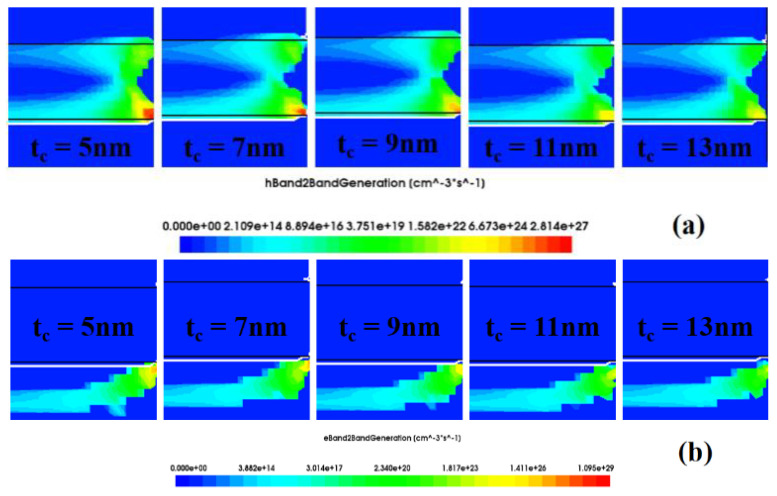
(**a**) The hole generation rate and (**b**) electron generation rate in the source and channel regions on the left side of the DM−SSTGTFET biosensor under different thicknesses of nanogap.

Figure 6 shows a cross-section of a part of the source region and the drain region near the left side of the gate. This figure shows the hole generation and electron generation for band–band tunneling at different nanogap thicknesses. As the thickness of the nanogap increases, the rate of hole and electron generation decreases. Therefore, the more difficult the biosensor is to turn on, the greater the threshold voltage and the greater the decrease in the on-state drain current. This is consistent with the conclusion drawn in Figure 5b.

### 3.3. Different Charged Biomolecules

**Figure 7:**
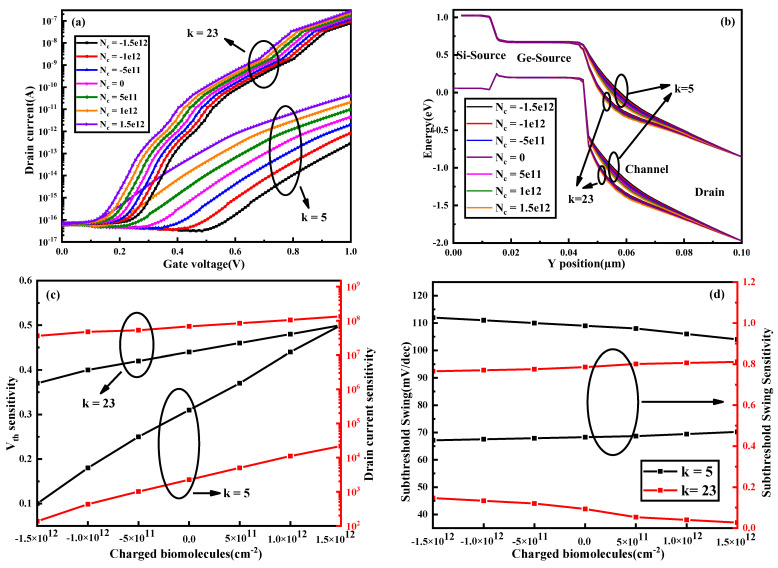
(**a**) Transfer characteristics, (**b**) energy band variation with respect to the *y*−axis and (**c**) *S_Vth_* and *S_d_* and (**d**) *SS* and *S_SS_* of the DM−SSTGTFET biosensor for different charged biomolecules at *V_d_* = 0.5 V, *V_g_* = 1 V and *t_c_* = 5 nm.

The biomolecules discussed above are all neutral molecules and are not charged. Therefore, it is necessary to consider different charged biomolecules. The different charge densities investigated in this paper are 5 × 10^11^ cm^−2^, 1 × 10^12^ cm^−2^ and 1.5 × 10^12^ cm^−2^. As shown in Figure 7, the smaller the k value, the greater the change in the transfer curve, energy band, threshold voltage sensitivity and drain current sensitivity under different charged biomolecules.

### 3.4. Partially Filled

**Figure 8:**
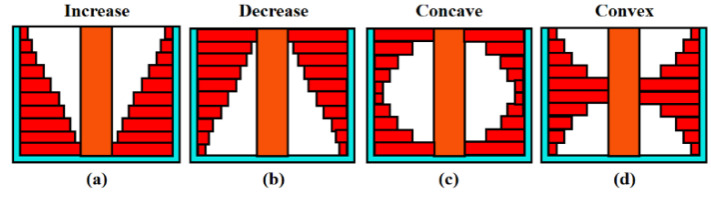
Distributions of partially filled nanogaps: (**a**) increase, (**b**) decrease, (**c**) concave (**d**) convex.

The results discussed above are all based on the complete filling of the nanogaps. Therefore, this section discusses the effect of partially filling the nanogaps on the sensitivity of the proposed biosensors. We mainly studied four distributions of biomolecules in the nanogaps. As shown in Figure 8, they are increase, decrease, concave, and convex distributions, respectively. In the two distribution methods “increase” and “decrease”, the height of the nanogap is 60 nm and it is divided into ten parts evenly. Therefore, the height of each small part is 6 nm. The thickness of the nanogap is 5 nm, which is also divided into ten evenly. Therefore, the minimum thickness of each small part is 0.5 nm, and they increase in increments of 0.5 nm. By calculation, the filled area is 55% of the total area of the nanogap. In the two distribution methods “concave” and “convex”, the height of each small part is 6 nm, which is the same as the previous two distribution methods. The minimum thickness of the small part is 1 nm, and they increase in increments of 1 nm. The maximum thickness of the small part is 5 nm. By calculation, the filled area is 60% of the total area of the nanogap.

**Figure 9:**
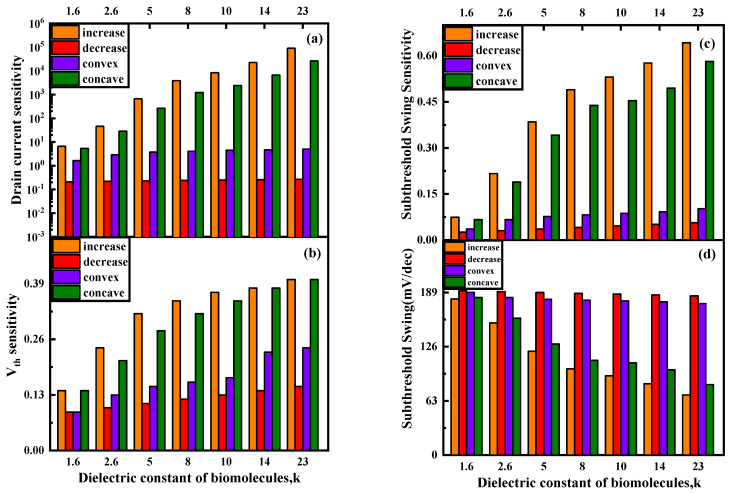
(**a**) Drain current sensitivity, (**b**) *V_th_* sensitivity, (**c**) subthreshold swing sensitivity and (**d**) subthreshold swing of DM−SSTGTFE biosensors for different k value and distribution at *V_d_* = 0.5 V, *V_g_* = 1 V and *t_c_* = 5 nm.

Figure 9 shows the changes in sensitivity of the proposed biosensors under different biomolecule fillings and different filling distributions. It can be seen from Figure 9 that as the dielectric constant of the biomolecules increases, the current sensitivity, threshold voltage sensitivity, subthreshold swing and subthreshold swing sensitivity of the biosensor all increase. This is consistent with the conclusion in Section 3.1. As shown in Figure 9a–c, the drain current sensitivity, threshold voltage sensitivity and subthreshold swing sensitivity have the largest change in the “increase” distribution, followed by the “concave” distribution, then the “convex” distribution, and then the “decrease” distribution, which has the smallest change. It can be seen from Figure 6 that electron tunneling and hole tunneling mainly occur near the bottom and the middle of the gate. Among them, the tunneling rate at the bottom of the gate is stronger. The “increase” filling form has the largest filling area near the bottom of the gate, so the sensitivity change is also the largest. The “concave” form has a slightly smaller area near the bottom of the gate, so the sensitivity change is second largest. The “convex” form has a larger filling area near the middle of the gate, while the “decrease” form has a larger filling area near the top of the gate. Therefore, the sensitivity of “convex” is greater than that of “decrease”.

### 3.5. Comparision with TFET-Biosensor

Many researchers have conducted research on TFET-based biosensors. This section compares the sensitivity of other TFET biosensors with the proposed structure. Table 2 shows a sensitivity data comparison between the proposed structure and other TFET biosensors. Among them, *I_on_*/*I_off_* is the ratio of the on-state drain current to the off-state drain current of the device. Through comparative analysis, it can be seen that the threshold voltage sensitivity and sub-threshold swing sensitivity of the DM-SSTGTFET biosensor are both the highest. At the same time, the current sensitivity and *I_on_*/*I_off_* are relatively high.

In short, the structure design of DM-SSTGTFET biosensor is reasonable, and the device has excellent tunneling and electrical characteristics. Therefore, the proposed biosensor has high sensitivity for the detection of biomolecules and has wide application prospects in the future.

## 4. Conclusions

In summary, through the research on the threshold voltage sensitivity, drain current sensitivity and subthreshold swing sensitivity of the DM-SSTGTFET biosensor, the proposed biosensor has high sensitivity for biomolecule detection in the field of biosensor applications. At the same time, the effects of different biomolecules in the nanogaps, different thicknesses of the nanogaps and different charges of the biomolecules were studied. The larger the dienlectric constant, the easier it is for the biosensor to detect the biological molecule. The smaller the nanogap between biomolecules, the easier it the detection. Positively charged biomolecules are easier to detect. These observations are significant for the detection of some specific biomolecules by biosensors and show that TFET biosensors have development potential.

## Figures and Tables

**Table 1:** Device parameters used for the simulation.

Parameter Name	Symbol	Value	Unit
Oxide thickness	T_ox_	2	nm
Drain height	H_d_	18	nm
Si source height	H_Si_	15	nm
Ge source height	H_Ge_	30	nm
Channel doping	N_c_	5 × 10^14^	cm^−3^
Source doping	N_s_	1 × 10^20^	cm^−3^
Drain doping	N_d_	1 × 10^18^	cm^−3^
Nanogap height	H_gap_	60	nm
Gate work function	Φ_MS_	4.4	eV

**Table 2:** Sensitivity comparison of the DM-SSTGTFET biosensor with other reported TFET biosensors.

	*S_Vth_*/*V*	*S_d_*	*S_SS_*	*I_on_*/*I_off_*
This work	0.46	10^8^	0.8	2.5 × 10^9^
Ref. [18]	0.29	-	0.17	10^8^
Ref. [22]	-	10^9^	-	9 × 10^8^
Ref. [23]	-	1 × 10^5^	-	1 × 10^6^
Ref. [25]	0.06	200	-	10^8^
Ref. [29]	-	300	0.5	1 × 10^9^
Ref. [31]	-	10^3^	0.2	3 × 10^9^
Ref. [32]	-	100	0.25	10^9^

## Data Availability

The data presented in this study are available on request from the corresponding author. The data are not publicly available due to confidentiality requirements.
